# Supplementary material for: HIVprotI: an integrated web based platform for prediction and design of HIV proteins inhibitors
Source: J Cheminform. 2018 Mar 9;10:12. doi: 10.1186/s13321-018-0266-y (PMC5845081; doi:10.1186/s13321-018-0266-y)
Supplement: Supplementary file 2 — Additional file 2. Source code of HIVProtI web server. [file 13321_2018_266_MOESM2_ESM.zip › HIVprotI_Source-code/hivprotI/submit_rt.php]

include("head.php");
?
  
  

#### Reverse transcriptase inhibitor prediction

Users can draw the structure of a desired chemical compund
and subsequently predict its percentage inhibition alongwith other properties.

|  |  |  |
| --- | --- | --- |
|  |  | **Help** - To load a molecule (.MOL file) click . - The   deletes clicked atoms. - The  deletes everything on the canvas. - For further instructions click here. - To screen a compound against multiple proteins simultaneously, click here. |

  

#### Select protein:

|  |  |  |  |  |  |  |  |  |  |
| --- | --- | --- | --- | --- | --- | --- | --- | --- | --- |
|  | Reverse transcriptase |  | Protease |  | Integrase |  | CHECK ALL |  |  |

  

Loading... Please wait (2-5 min)

  


include("foot.php");
?
